# Case Report: Congenital tuberculosis in an aborted dromedary camel fetus

**DOI:** 10.3389/fvets.2022.956368

**Published:** 2022-07-28

**Authors:** Shirish Dadarao Narnaware, Basanti Jyotsana, Rakesh Ranjan, Ved Prakash, Shyam Sundar Choudhary, Artabandhu Sahoo

**Affiliations:** ICAR-National Research Centre on Camel, Bikaner, Rajasthan, India

**Keywords:** camel, congenital tuberculosis, *Mycobacterium tuberculosis*, pathology, vertical transmission, abortion

## Abstract

Tuberculosis (TB) is a serious public health problem worldwide, especially in tropical developing countries. Nevertheless, reports on congenital TB in humans and animals are extremely rare. In this study, abortion was reported in an 8-year-old she-camel at the 9th month of gestation. The she-camel appeared healthy in clinical examination, had a good body condition score, normal appetite, and had no signs of respiratory disease and fever. The expelled placenta was dark red-colored, thickened, and edematous with multifocal to coalescing ecchymotic hemorrhages on the allantoic surface. The striking finding was multiple, white-yellow, solid nodular lesions in the fetal lung, the pleura, and the liver. On histopathology, typical granulomatous lesions were detected in the lung and the liver characterized by caseous necrosis surrounded by lymphocyte and macrophage infiltration and concentric layers of fibrosis. The Ziehl-Neelsen staining detected scarce acid-fast bacilli in lung and liver tissues. The DNA extracted from tubercular lesions from the lung and liver showed amplification of the IS6110 region of the *Mycobacterium tuberculosis* complex by PCR. The sequencing and phylogenetic analysis revealed a close association of these sequences with *Mycobacterium tuberculosis*. The she-camel was detected positive for a single intradermal tuberculin test performed 24 h after abortion. This is the first report on congenital TB caused by *M. tuberculosis* in a dromedary camel fetus with a possible vertical transmission.

## Introduction

Tuberculosis (TB) is an ancient chronic contagious granulomatous disease having zoonotic and economic potential all over the world, especially in tropical developing countries. As per the WHO, 1.3 million deaths were reported as caused by TB globally in 2020, and TB mortality has been more severely impacted by the COVID-19 pandemic than HIV/AIDS (1). In affected countries, the disease has an important socio-economic and public health-related impact and also represents a serious constraint in the trade of animals and their products (2). The emergence of multidrug-resistant TB is a global threat and a big challenge for effective control of the disease all over the world.

In dromedary camels, Mycobacteria belonging to the *Mycobacterium tuberculosis* complex (MTBC) have been frequently isolated; however, *Mycobacterium bovis* was reported as the most common etiological agent (3, 4). The most frequent clinical signs of camel TB are chronic weight loss, weakness, and lethargy; nevertheless, respiratory signs and fever were also recorded infrequently (3, 5). The lesions usually form in the lungs and the associated lymph nodes, and hematogenous or lymphatic spread can occur to the other organs (6).

The transmission of TB in camels may occur through contact with infected camels or other livestock and the route of infection are mainly through inhalation or ingestion (6). Although abortions and infertility due to TB lesions in the uterus have been sporadically reported in cattle (7), the intrauterine infection of TB is not yet reported in camels. This study describes a case of congenital TB caused by *M. tuberculosis* in an aborted dromedary camel fetus.

## Case description

An 8-year-old Kachchi breed of dromedary camel was presented with abortion after the 9th month of gestation during her 2nd pregnancy. This camel belonged to a herd comprised of 350 dromedary camels in the Thar desert of Rajasthan, India. The herd is maintained in a semi-intensive husbandry system, mostly in outdoor facilities, and fed with a mix of pellet feed, hay, and *ad libitum* water. A history of sporadic occurrence of TB has been reported in this herd. Clinically, the aborted she-camel appeared healthy with a good body condition score and normal appetite and did not show any respiratory symptoms and fever at the time of the abortion. The she-camel was tested negative for brucellosis on Rose Bengal Plate Test. However, she tested positive in a single intradermal tuberculin test performed a day after abortion, showing marked swelling and a 2-fold increase in skin thickness at the injection site.

**Figure 1:**
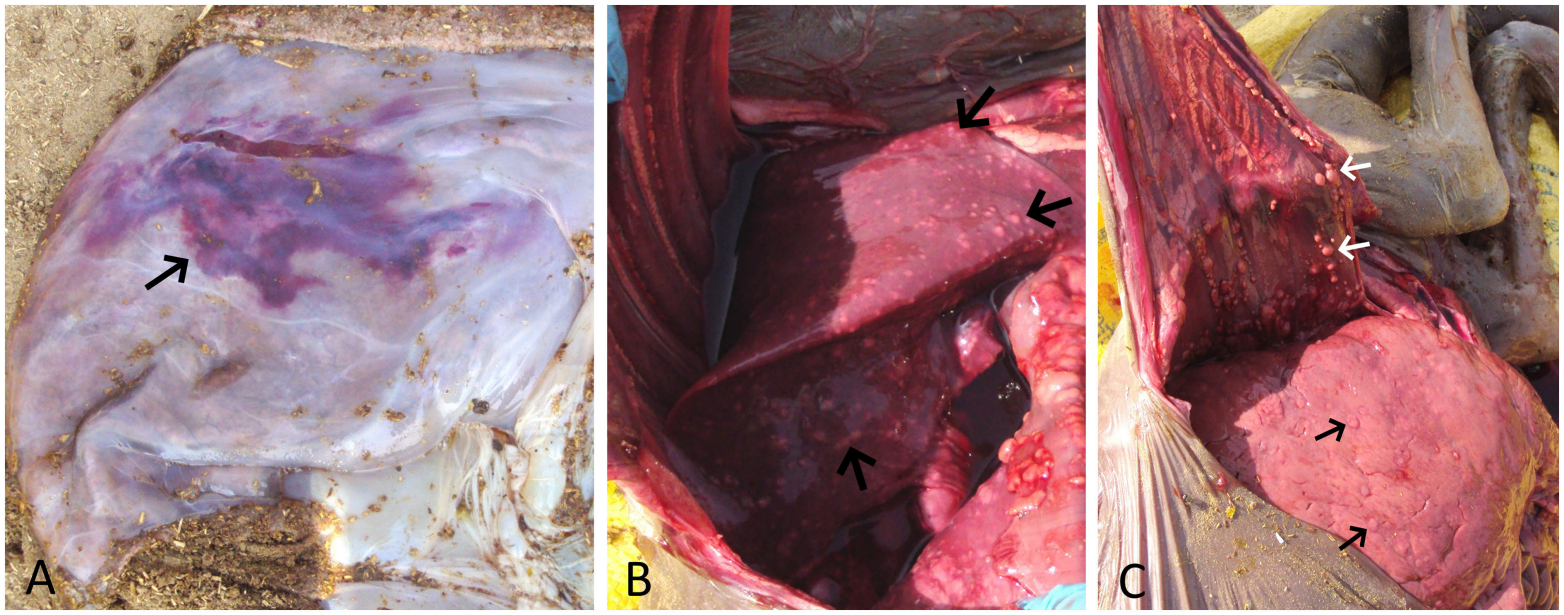
**(A)** Placenta showing edema and a large area of ecchymotic hemorrhage (arrow) on the allantoic surface. **(B)** Fetal lung showing congestion and multiple yellow-white tubercle nodules scattered throughout its surface (arrow). **(C)** Enlarged and congested fetal liver showing multiple yellow-white tubercle nodules (black arrow). Also, note multiple tubercle nodules adhered to the inner surface of the thoracic cavity (white arrow).

After the abortion, the placenta and aborted fetus were examined for gross lesions. The expelled placenta was thickened, edematous, and multifocal to coalescing ecchymotic hemorrhages on the allantoic surface (Figure 1A). The fetus showed generalized subcutaneous edema, congestion, and a moderate amount of sero-hemorrhagic fluid in the abdominal and thoracic cavity along with generalized congestion of all internal organs, which is likely due to autolysis and hemoglobin imbibition. The striking finding in the fetus was the presence of multiple, white-yellow, solid nodules scattered over the lung, the pleura, and the liver which measured from 2 to 15 mm in diameter (Figure 1B). The lung was collapsed, severely congested, and had multiple small white-yellow tubercle nodules scattered on all lobes. These nodules were also found attached to the pleura and inner surface of the rib cage. The liver was found enlarged considerably, congested, and had multiple tubercle nodules (Figure 1C). The other organs viz., the heart, the spleen, the kidney, and the intestines showed severe generalized congestion without any evidence of tuberculous lesions.

**Figure 2:**
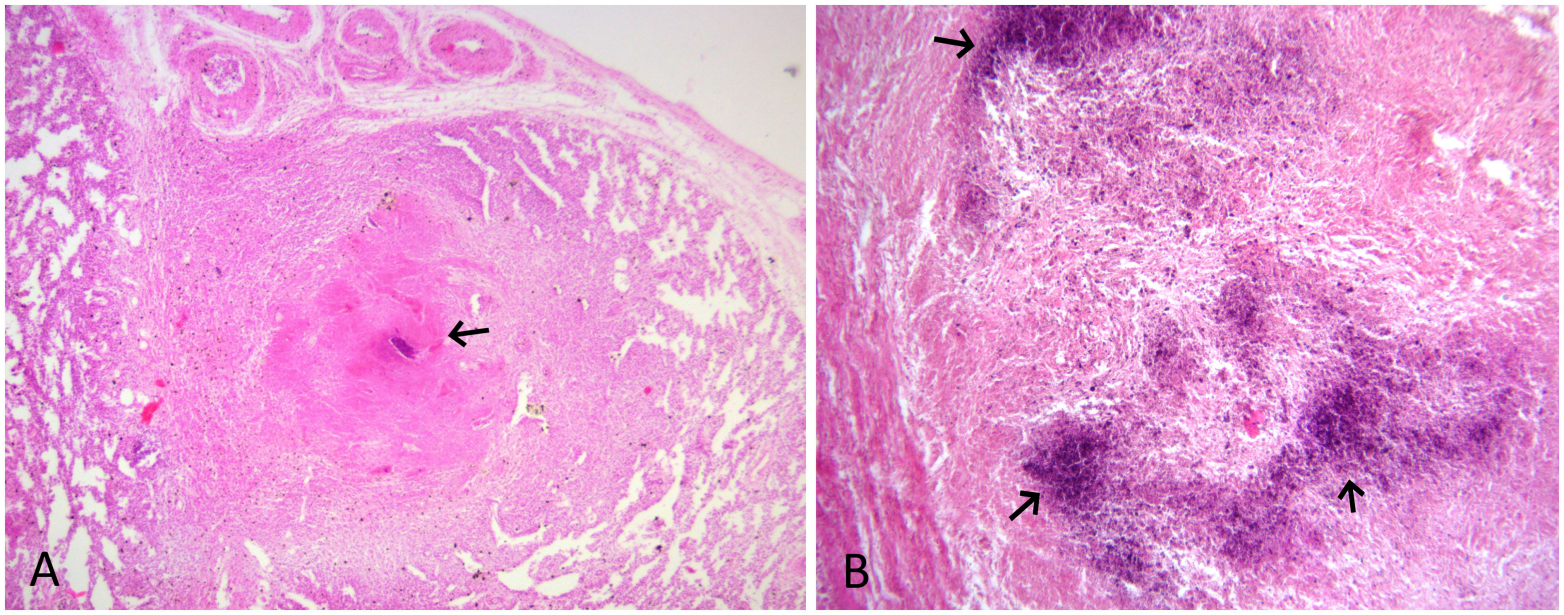
**(A)** HE-stained lung section showing granuloma with mineralization and caseous necrosis (arrow) surrounded by scattered lymphocyte and macrophage infiltration, and concentric layers of fibrosis. HE x 100. **(B)** HE-stained liver section showing dark blue areas of mineralization (arrow) and macrophage and fibrous tissue infiltration. HE x 200.

**Figure 3:**
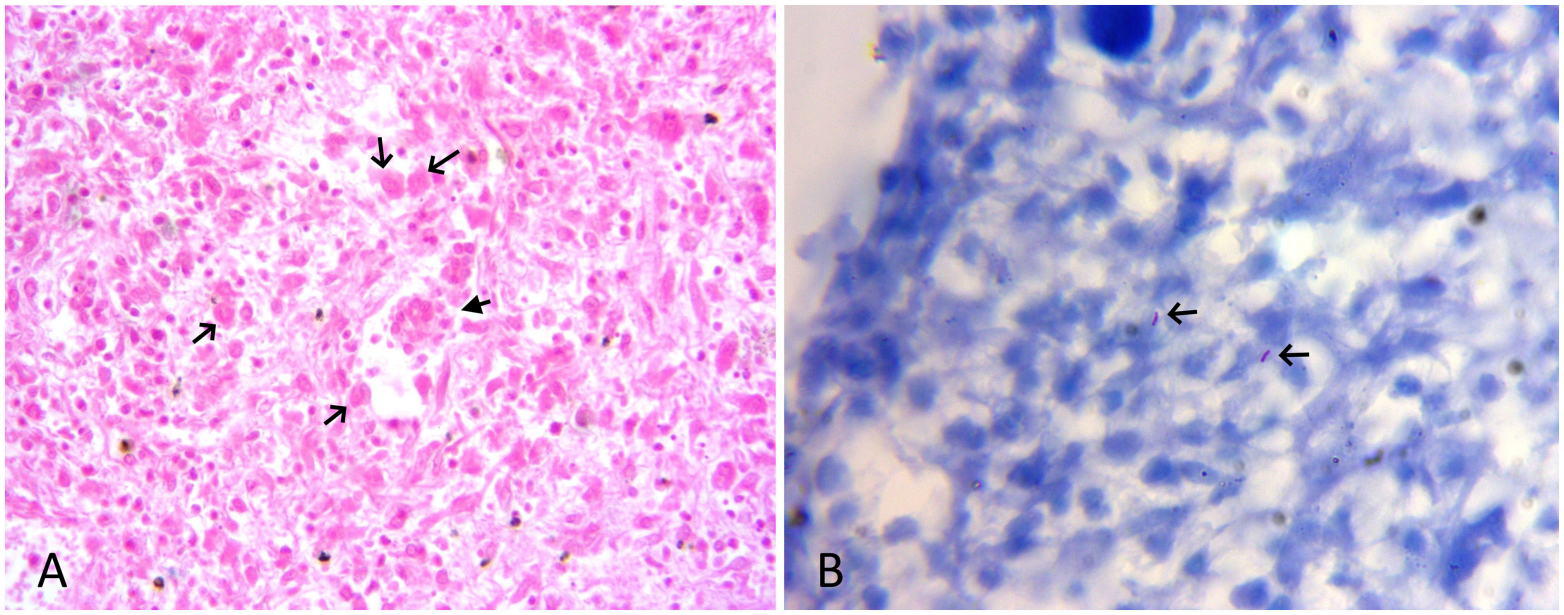
**(A)** HE-stained lung section showing scattered macrophages (black arrow), few lymphocytes, and a multinucleate giant cell (filled arrow). HE x 400. **(B)** ZN-stained section of liver showing sparse acid-fast bacilli (arrow). ZN x 1,000.

The tissue samples suspected to have TB lesions, such as the lung, the liver, and the placenta, were collected in 10% neutral-buffered formalin for histopathology, as well as in sterile vials for DNA extraction. For histopathology, tissues after fixation were embedded in paraffin, cut into 4-μm-thick sections and stained with hematoxylin and eosin. Selected sections were also subjected to Ziehl-Neelsen (ZN) staining. On histology, the lung and liver sections showed typical granulomatous lesions characterized by a central area of caseous necrosis and mineralization surrounded by scattered lymphocytes, macrophages, and/or occasional giant cells, and concentric layers of fibrosis (Figures 2A,B, 3A). Scarce acid-fast bacilli were observed on ZN-stained sections of the lung and the liver (Figure 3B). Histopathology of the placenta showed normal physiologic and/or autolytic changes of mineralization of the chorionic epithelium, necrosis of villous stroma, and hyperemic blood vessels. However, granulomatous inflammation and acid-fast bacilli were not observed.

Tissues (placenta, fetal lung, and liver) were processed for DNA extraction using the PureLink™ Genomic DNA Mini Kit (Invitrogen). The DNA was subjected to PCR amplification of a 245bp region of IS6110 sequence specific for the MTBC, using primer pairs INS1 (5′-CGTGAGGGCATCGAGGTGGC-3′) and INS2 (5′-GCGTAGGCGTCGGTGACAAA-3′) (8). Briefly, a 25 μl reaction was prepared using 12.5 μl Gotaq^®^ green master mix (Promega), 1 μl each primer (10 picomoles), 5 μl of DNA, and 5.5 μl of nuclease-free water. The cycling conditions used were initial denaturation at 94°C for 5 min, followed by 35 cycles of denaturation at 94°C for 1 min, annealing at 65°C for 1 min, and extension at 72°C for 1 min. This was followed by a final extension step of 72°C for 10 min. The PCR-amplified products were visualized in 2% agarose gel and the purified PCR products were subjected to nucleotide sequencing for the IS6110 gene using Sanger sequencing based on the chain-terminating dideoxynucleosides method (Eurofins, India). These sequences were deposited in NCBI GenBank (accession numbers: MW393780 and OL436218) and aligned with the published sequences for phylogenetic analysis using the ClustalW tool and the Maximum Composite Likelihood method (9). This analysis involved 30 nucleotide sequences, and the evolutionary analyses were conducted in MEGA X (10). The DNA extracted from the lung and the liver showed amplification of the IS6110 region of MTBC by PCR (Supplementary Figure 1). The sequencing and phylogenetic analysis revealed that sequences in this study clustered with *M. tuberculosis*.

On a managemental aspect, this she-camel was isolated and maintained away from the herd after abortion. After 1 year of isolation, the she-camel exhibited the clinical signs of chronic infection, including poor appetite, weakness, and progressive emaciation. Eventually, this she-camel died after 7 months of exhibiting symptoms. Considering the clinical history and the safety of the personnel, neither necropsy nor tissue evaluation was performed on this she-camel. Instead, the carcass was immediately disposed of by deep burying.

## Discussion

Based on the pathological findings and detection of the MTBC genome from the fetal tissues, the case was etiologically diagnosed as fetal systemic mycobacteriosis caused by *M. tuberculosis* infection. The systemic TB lesions in aborted fetuses characterized by granulomatous inflammation were comparable with earlier reports of systemic mycobacteriosis in an aborted mare (11) and congenital TB in a newborn calf (12), suggesting the vertical transmission of TB bacilli from the infected dam to the fetus in the dromedary camel. This is known as the first report of a congenital form of TB in camels. The congenital forms of the disease can occur when the disease involves the dam's genital tract or placenta (12). In such cases, TB bacilli are introduced into the fetus hematogenously *via* the umbilical vein, or *via* infected amniotic fluid ingested or aspirated *in utero* or at birth (13, 14). The significant lesions in the chorionic epithelium of the placenta can also be responsible for inadequate nutrition or fetal oxygenation resulting in fetal anoxia and abortion (15). However, granulomas or foci of caseous inflammation were absent in the placenta, which is intriguing. Nevertheless, the presence of mineralization, necrosis, cell desquamation, and minimal inflammatory cells observed in the placenta could be a resemblance to physiologic or autolytic changes observed in cattle placenta (16).

Although emaciation, weakness, and respiratory symptoms are commonly reported in TB-affected camels (3), abortion due to systemic fetal mycobacterial infection is not yet recorded. However, this she-camel exhibited no apparent clinical signs apart from abortion. It is possible that this she-camel was in the early stage of infection or had latent TB infection with minimal lesions and no apparent symptoms. As TB has a very long incubation period, physiological stresses, such as pregnancy and poor nutrition state could have triggered the activation of mycobacterial infection after abortion (17, 18). Moreover, the increased susceptibility to infections has been observed in periparturient cows mainly due to deficient systemic and local immune responses around parturition (18). It was suggested that bacterial invasion of the chorionic surface and subsequent hematogenous spread might be responsible for causing the fetus' systemic infection in equine species (11). Since camel placenta is epitheliochorial and resembles equine placenta, and hence this infectious route can also be possible in camels.

Since the clinical signs of TB in camelids often go unnoticed, and they are asymptomatic until the disease is advanced (17), therefore, nomadic people, who are in close association with the rearing and handling of camels are, at high risk of being infected. Given this, camel TB is a disease of concern from the point of its economic and zoonotic significance, especially in countries where camel has special cultural and economic importance. Few countries, such as Australia, some Caribbean islands, and parts of South America, eradicated bovine TB using a test-and-slaughter policy, which has drastically reduced the incidence of disease in both animals and humans (19). In addition, to control the TB spreading between camels and humans in endemic areas, the focus should be given to regular surveillance using rapid diagnostic tests for earliest case detection, segregation of the suspected animals, and educating the camel farmers about the risk of infection.

In conclusion, a congenital transmission of mycobacteria is evident in camels. Also, a camel could be a potential source of latent TB infection. Hence, regular screening of camels for mycobacterial infection is suggested for minimizing the risk associated with the spread of TB in endemic areas.

## Data availability statement

The datasets presented in this study can be found in online repositories. The names of the repository/repositories and accession number(s) can be found below: https://www.ncbi.nlm.nih.gov/genbank/, MW393780 and https://www.ncbi.nlm.nih.gov/genbank/, OL436218.

## Ethics statement

Ethical review and approval was not required for the animal study because the case was natural infection and samples were collected from dead animal.

## Author contributions

SN designed the experiments and prepared the manuscript. BJ, RR, VP, and SC collected the samples and reviewed the manuscript. AS supervised the project. All authors have read and approved the final version of the manuscript.

## Conflict of interest

The authors declare that the research was conducted in the absence of any commercial or financial relationships that could be construed as a potential conflict of interest.

## Publisher's note

All claims expressed in this article are solely those of the authors and do not necessarily represent those of their affiliated organizations, or those of the publisher, the editors and the reviewers. Any product that may be evaluated in this article, or claim that may be made by its manufacturer, is not guaranteed or endorsed by the publisher.
