# Supplementary material for: Case Report: Congenital tuberculosis in an aborted dromedary camel fetus
Source: Front Vet Sci. 2022 Jul 28;9:956368. doi: 10.3389/fvets.2022.956368 (PMC9368318; doi:10.3389/fvets.2022.956368)
Supplement: Supplementary file 1 [file Data_Sheet_1.PDF]

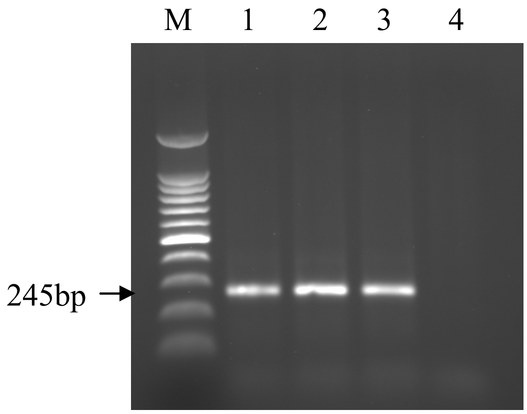

Supplementary Figure 1. PCR amplification and agarose gel electrophoresis of *M. tuberculosis* IS6110 gene. Lane M: 100bp DNA ladder; 1: Lung DNA; 2: positive control (*M. tuberculosis* positive DNA); 3: Liver DNA; 4: Placenta DNA.
